# Exposure rate of VZV among women attending antenatal care clinic in Sri Lanka - a cross sectional study

**DOI:** 10.1186/s12879-017-2725-x

**Published:** 2017-09-16

**Authors:** Saluwadana Walawwe Pavithra Lakmini Daulagala, Faseeha Noordeen, Mohamed Mukthar Fathina Fara, Chathura Rathnayake, Kapila Gunawardana

**Affiliations:** 1 Department of Microbiology, Faculty of Medicine, University of Peradeniya, Peradeniya, Sri Lanka; 2 Department of Gynecology and Obstetrics, Faculty of Medicine, University of Peradeniya, Peradeniya, Sri Lanka

**Keywords:** Antenatal, Anti-VZV IgG, Epidemiology, Sero-prevalence, Sri Lanka, Varicella

## Abstract

**Background:**

Varicella or chickenpox was not a notifiable disease until 2005 in Sri Lanka and only a few studies have been conducted on the epidemiology of VZV infection in the country. The anti­VZV IgG sero-prevalence among antenatal women is extremely limited and thus a selected group of antenatal clinic attendees were chosen to determine the exposure rate to VZV infection.

**Methods:**

Women attending the antenatal clinic at Teaching Hospital, Peradeniya, Sri Lanka were selected for the study and 3 mL of venous blood was collected from 181 participants and the demographic data was obtained through a pre­tested questionnaire. Sera of the women were then tested for the presence of anti­VZV IgG using ELISA (HUMAN Diagnostics, Germany). Data was analysed using the SPSS statistical software for Windows, Version 12.0.

**Results:**

Of the 181 antenatal women who took part in the study, 141 were positive for anti­VZV IgG giving a sero-prevalence of 77.9% for the past exposure to VZV. Of the 141 anti­VZV IgG positive women, 43.3% (*n* = 61) were from urban, 41.8% (*n* = 59) were from rural and 14.9% (*n* = 21) were from estate populations (an ethnic population living in small settlements in the tea estates whose ancestors were brought from India during the British colonial period to work in the tea plantations in Sri Lanka). Out of the 88 antenatal women with a positive history for varicella, 85 (96.6%) were positive for anti-VZV IgG. The highest number of anti­VZV IgG positivity was seen in the 31–35 age group, which was 85.0% of the total number of antenatal women included in that category. An increase in the anti­VZV IgG sero-prevalence with increasing age was also noted in the study sample.

**Conclusion:**

Exposure rate of VZV infection as confirmed by anti­VZV IgG in the present study sample of antenatal women was 77.9%. Age specific, population based future sero-prevalence studies should be conducted in Sri Lanka to understand the anti-VZV IgG status in the country.

## Background

Varicella zoster virus (VZV) is a ubiquitous virus that infects humans. VZV causes a highly contagious disease called varicella or chickenpox, which has an attack rate of 65 to 85% in susceptible individuals followed by household exposure [1, 2]. Although varicella is a self-limiting disease, the risk of hospitalizations and complications following varicella among pre-school and school children is higher than that was reported a decade ago [3].

VZV infections occur worldwide but the epidemiology differs greatly between tropical and temperate climates. In temperate countries, children are predominantly affected by VZV infections and the sero-conversion to anti-VZV IgG occurs usually during late childhood and thus adults show up to 90 to 95.0% serological evidence for the past exposure to the virus [4]. Avaccine naive 1–17 year old German study sample showed an increasing sero-prevalence for anti-VZV IgG with 60.0% and >90.0% of the children showing immunity to varicella by theage of4 and 9 years, respectively [5]. Moreover, in temperate countries VZV infection rate is high during winter and early spring [6]. The epidemiology of varicella is partly understood in tropical and subtropical regions. Differences in exposure rates to VZV infection in different age groups have been noted with different hypotheses [7, 8]. The exposure to the virus at late childhood or young adulthood causes high morbidity in the high school and university students and young work force in tropical nations. The climatic factors like humidity, socio-economic conditions and cultural practices appear to play a role for the differences in the exposure to the virus in the tropics [9, 10].

In tropical countries, the VZV infections are common in adolescents and adults [6]. The clinical severity of the disease in adults is higher than that in children, while it could be fatal in immunocompromised and elderly individuals. These findings suggest that adults in the tropical countries maybe at high risk for acquiring VZV infection due to late sero-conversion to anti-VZV IgG and thus experiencing morbidity and a mortality rate of up to15.0% [11]. Based on a recent South Korean study, incorporating the universal varicella vaccine to the National Immunization Programme since 2005 has not decreased the incidence of varicella, 22.5 per 100,000 persons in 2006 to 73.2 per 100,000 persons in 2013 [12]. However, a difference in the anti-VZV IgG sero-prevalence rate is noticeable even among different tropical countries. Some of the tropical and subtropical countries such as United Arab Emirates, Saudi Arabia and Iran show sero-prevalence of more than 80.0% against anti-VZV IgG while other tropical countries like Singapore, Pakistan, India and Sri Lanka show lower sero-prevalence rates of 40–60.0%, making a significant proportion of adults susceptible to VZV in these countries [13–15]. On the other hand, these countries, Pakistan, India and Sri Lanka, with higher susceptibility to VZV infection than other tropical countries are located closer to the equator.

Varicella was not a notifiable disease until 2005 and only a few studies have been conducted on the epidemiology of VZV in Sri Lanka. Based on a study carried out in a selected urban and rural population in Colombo, none of the children below 5 years in the rural area had detectable anti-VZV IgG. Only 10.0% of the children in the urban population had sero-positivity for anti-VZV IgG. In the same study, a sero-positivity of 17.0% and 24.0% for anti-VZV IgG was observed among children below 15 years in urban and rural populations, respectively. In those aged 60 years, only 50.0% in the rural population were immune to VZV whereas in the urban population, 78.9% were immune to VZV [16].

There is limited data on the anti­VZV IgG sero-prevalence among antenatal women in the country. The economic drain posed by varicella and its complications are currently not studied and the incidence data on varicella are sparse in Sri Lanka. Varicella vaccine is not included in the National Immunization Programme in Sri Lanka [17]. However, the vaccine is available on requestfor those who are at risk of acquiring varicella including the medical and nursing students and the military trainees with negative history of varicella. Age specific community based studies are mandatory at district levels to find out the susceptibility rate among different age groups, which would be helpful in identifying the target population for vaccination in the country.

The objective of the present study was to determine the exposure rate to VZV infection in a selected group of antenatal clinic attendees at a Teaching Hospital in Sri Lanka. The study also aimed to determine theexposure rate to VZV infectionin urban, rural and estate communities; to determine the exposure rate to VZV infection in antenatal women in different age categories; to assess the suitability of using the past history of varicella as a predictor of immunity against VZV infection in the study sample.

## Materials and methods

This cross-sectional study was conducted from December 2015 to February 2016. The sample size for the study was determined using the 92.0% prevalence rate for anti-VZV IgG observed for a group of antenatal women in the tropical Australia [18]. Based on the formula used to determine the prevalence rates for health studies, a sample size of 113 was derived with 92.0% prevalence rate for anti-VZV IgG and thus we decided to recruit a study sample of more than 113. Demographic data and blood samples were collected from 181 antenatal women attending two of the antenatal clinics at Teaching Hospital, Peradeniya, Sri Lanka between18–45 years of age. Samples were collected without excluding any attendee, who consented to take part in the study. Demographic data and past history of varicella in the study sample were recorded using a questionnaire. Even if most in the study sample could recall whether they havehad varicella or not in the past, 41 antenatal women were doubtful of their past history of varicella.

The information sheet and consent form were given to the participants prior to filling the pre-tested questionnaire and obtaining the blood sample. Blood samples were kept in a cool box and transported to the Virology Laboratory, Faculty of Medicine, University of Peradeniya. The samples were centrifuged at 3000 rpm for 20 min at room temperature to separate the serum. Sera were separated into properly labeled eppendorf tubes and stored at −20 °C freezer until tested for anti-VZV IgG. All the serum samples of individual patients were tested for the presence of anti-VZV IgG using a standard commercially available VZV IgG ELISA (HUMAN, Germany) with a sensitivity and specificity of more than 90.0% for detecting anti-VZV IgG. During the laboratory testing of the serum samples for anti-VZV IgG, no equivocal results were obtained for any of the 181 sera tested.

Data was entered into SPSS, a statistical software for Windows, Version 12.0 for analysis. Results were described through measures of central tendencies including mean, standard deviations and proportions. The relationship between categorical variables such as past history of varicella andVZV immune status and, age and VZV immune status was checked using the Chi square test.

## Ethical considerations

Ethical clearance (PGIS/2016/EC22) for the study was obtained from the Ethical Review Committee of the Post Graduate Institute of Science (PGIS), University of Peradeniya.

## Results

The antenatal attendees, who took part in this study were from diverse socio-economic conditions and religiously diverse backgrounds. Most of them were unemployedand these women were mostly from middle class and from the estate sector families of Sri Lanka. Of the 181 antenatal women tested in the present study, 141 (77.9%) showed anti- VZV IgG positivity. Of the 141 anti-VZV IgG positive antenatal women, 61 (43.3%) were from urban, 59 (41.8%) were from rural and 21 (14.9%) were from estate populations.

**Fig. 1:**
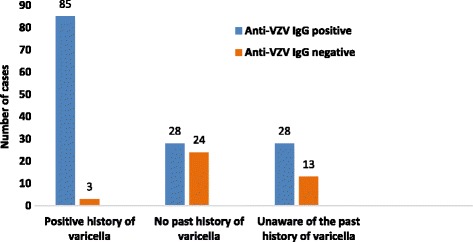
Relationship between past history of chickenpox and anti-VZV IgG status in the study population of antenatal women

From the 88 antenatal women with a positive past history for varicella, 85 were positive for anti-VZV IgG, which is 96.6% from the total number of antenatal women, who reported a past history of varicella. Of the 52 antenatal women with no a past history of varicella, 28 (53.8%) showed antibodies against VZV. Of the 41 antenatal women who were unaware of their past history for varicella, 28 (68.3%) were positive for anti-VZV IgG (Fig. 1).

**Fig. 2:**
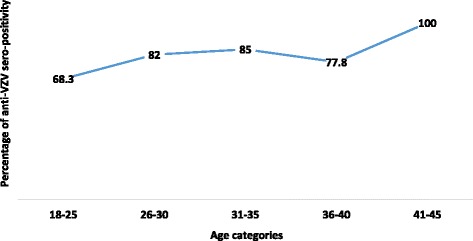
Anti-VZV IgG sero-positivity with increasing age in the study population of antenatal women

The study population was divided into 5 age categories as shown in the Table 1. The highest sero-prevalence of anti­VZV IgG positivity was seen in the 31–35 age group, which was 85.0% of the antenatal women who were included in that age category. The advancement in the age and the anti­VZV IgG positivity was positively associated as there was a rise in the anti­VZV IgG sero-prevalence with the increase in the age (Fig. 2).

**Table 1:** Anti-VZV IgG status in different age categories of antenatal women

			Anti-VZV IgG		Total
			Anti-VZV IgG negative	Anti- VZV IgG positive	
Age categories	18–25	Number	19	41	60
		% within category	31.7%	68.3%	100.0%
	26–30	Number	11	50	61
		% within category	18.0%	82.0%	100.0%
	31–35	Number	6	34	40
		% within category	15.0%	85.0%	100.0%
	36–40	Number	4	14	18
		% within category	22.2%	77.8%	100.0%
	41–45	Number	0	2	2
		% within category	0%	100.0%	100.0%
Total		Number	40	141	181
		% within category	22.1%	77.9%	100.0%

## Discussion

Of the 181 antenatal women (age = 18–45 years) included for the current study, the majority were positive for anti-VZV IgG, making more than ¾^th^ of the study population immune to VZV. In contrast to the current study findings, a study carried out in the Colombo District had less than ½ of the females in the child bearing agewere positive for anti-VZV IgG [16]. On the other hand, the new entrant students of the University of Peradeniya showed 49.1% sero-prevalence for anti-VZV IgG [19] and a group of adolescents from the Kandy District showed a sero-prevalence of 34.0% for anti-VZV IgG [20]. Thus, the sero-prevalence rate noted for anti-VZV IgG in the current study showed a considerable increment from previous studies done from 1999 to 2004 [16, 19, 20], however, the data on VZV sero-prevalence in Sri Lanka is limited in the past decade. Apart from the difference in the time at which past studies were conducted from now, the age groups covered in those studies may also be accountable for the discrepancies in the anti-VZV IgG status between those studies and the current study.

Of the 141 anti­VZV IgG positive antenatal women, 43.3% (*n* = 61) were from urban, 41.8% (*n* = 59) were from rural and 14.9% (*n* = 21) were from estate populations and these proportions when compared to a previous study in a group of adolescents in the Kandy District, sero-prevalence of anti-VZV IgG in the urban population (53.0%) had similarity to that noted in the current study. However, rural (17.0%) and estate communities (31.0%) had differences in sero-prevalence of anti-VZV IgG between the two studies [20]. Some other studies suggest that a delayed onset of VZV infection in rural tropics is purely a rural phenomenon and the degree of social disintegration among children might result in less opportunity for exposure in rural populations [21]. In other words, social integration must be encouraged from childhood not only to create an understanding and mutually respecting societies but also to get exposure to infections like varicella, which if acquired in childhood goes mild without complications with the development of lifelong immunity against the virus. However, in the current study, difference in the sero-prevalence was not significantly different between the antenatal women from urban and rural communities. This might be due to the age group tested by the current study having participants from 18 to 45 yearsallowing them to have exposure to VZV infection and thus sero-conversion to anti-VZV IgG.

When considering the impact of the past history of varicella, 85 out of the 88 antenatal women (97.0%) who had a past history for varicella were positive for anti-VZV IgG. These women (age = 18–45 years) had a better understanding and recalling power of the clinical history of varicella than those recruited for the previous studies in Sri Lanka, making a positive past history a more reliable indicator to predict immunity against varicella. Of the 52 antenatal women without a past history forvaricella, 28 (53.8%) were positive for antibodies against VZV and this might be due to subclinical VZV infection causing the anti-VZV IgG positivity. Similar findings have been reported by a Canadian study where 62.0% of population with negative or unknown history turn out to be anti-VZV IgG positive claiming that in the temperate climates the anti-VZV IgG sero-positivity is high and thus that acquired through subclinical infections would also be high [22]. Of the 41 (29.1%) antenatal women who were unaware or couldnot recall a past history of varicella, 28 (68.3%) were positive for anti-VZV IgG. This may be due to the lack of knowledge about varicella or acquiring the infection at a very early age and thus could not recall. In a study carried out in Colombo District, 28.0% of the study population with a self-reported history of varicellaturned out to be sero-negative for VZV IgG [16]. Based ona study carried out among adolescents in Kandy District,only 53.0% of those with a self-reported history of varicellashowed positivity for anti-VZV IgG [20]. Compared to the findings of the previous studies, in the present study, exposure to VZV recorded on the self- reported history of varicellaappears to be a reliable predictor ofimmunity.

When considering the relationship between anti-VZV IgG sero-prevalence and age of the antenatal women, the highest number of positive individuals was recorded in the 31–35 age group, which is 85.0% of the antenatal attendeesincluded for that age category. An increase in the anti­VZV IgG sero-prevalence with the age was noted among the antenatal women. However, the study population was not evenly distributed among the age categories and this might be the reason for the slight drop in the anti-VZV IgG positivity in women agedbetween 36–40 years. Due to limited funds this study was carried out using a sample size of 181 antenatal attendees but further studies should be carried out to confirm the consistency as well as changes in the anti-VZV IgG data among antenatal women in Sri Lanka.

## Conclusions

The current study identifieda relatively higher past exposure rate of ~ 78.0% to VZV in a vaccine naïve group of tropical antenatal women. This finding explains the limited number of neonatal chicken pox cases in a country with lesser exposure rates recorded for adolescents and adults in the last decade. Furthermore, age specific, population based sero-prevalence studies should be conducted in Sri Lanka to understand the anti-VZV IgG status in the country to incorporate VZV immunization to avoid the severity of adulthood varicellaand its complications.

## Abbreviations

CVS: Congenital varicella syndrome
ELISA: Enzyme-linked immunosorbent assay
IgG: Immunogolbulin G
VZV: Varicella zoster virus
